# Addition of New Androgen Receptor Pathway Inhibitors to Docetaxel and Androgen Deprivation Therapy in Metastatic Hormone-Sensitive Prostate Cancer: A Systematic Review and Metanalysis

**DOI:** 10.3390/curroncol29120747

**Published:** 2022-12-04

**Authors:** Francesco Fiorica, Consuelo Buttigliero, Daniela Grigolato, Marco Muraro, Fabio Turco, Fernando Munoz, Marcello Tucci

**Affiliations:** 1 Department of Radiation Oncology and Nuclear Medicine, AULSS 9 Scaligera, 37045 Verona, Italy; 2 Department of Oncology, University of Turin, San Luigi Gonzaga Hospital, 10093 Torino, Italy; 3 Radiation Oncology TomoTherapy Center, Hospital of Aosta, 11000 Aosta, Italy; 4 Department of Medical Oncology, Cardinal Massaia Hospital, 14100 Asti, Italy

**Keywords:** triplet therapy, hormone-sensitive prostate cancer, high volume metastatic disease, de novo metastatic disease, systematic review, metanalysis

## Abstract

In recent years, significant changes have occurred in metastatic hormone-sensitive prostate cancer (mHSPC) management, where docetaxel and new androgen receptor pathway inhibitors (ARPI) have been shown to improve overall survival (OS) compared to androgen deprivation therapy (ADT). Recent data could once again radically change mHSPC treatment. PEACE-1 and ARASENS trials demonstrated a survival benefit of the addition of ARPI to docetaxel and ADT combination (triplet therapy), compared to docetaxel and ADT. With multiple options to choose from, it is crucial to identify the patients who would benefit most from triplet therapy. In this meta-analysis, we evaluated the activity of the triplet therapy versus docetaxel plus ADT in mHSPC. A systematic review of PubMed/Medline, Embase, and the proceedings of major international meetings was performed. Five RCTs fulfilled the inclusion criteria. PEACE-1 and ARASENS studies reported disease-free survival (DFS) and OS. Post hoc analysis of three other trials evaluated the combination of ARPI, docetaxel and ADT. Globally, 2538 patients were included (1270 triplet therapy; 1268 docetaxel + ADT). Triplet therapy was associated with improved OS (hazard ratio (HR) 0.74; 95% confidence interval (CI), 0.66–0.83, *p* < 0.00001). A statistically significant benefit was shown in high-volume mHSPC patients (HR 0.76; 95% CI 0.59–0.97, *p* = 0.03) and in patients with de novo metastatic disease (HR 0.73; 95% CI, 0.64–0.82, *p* < 0.00001). The addition of ARPI to standard therapy was associated with DFS improvement (HR 0.41; 95% CI, 0.35–0.49, *p* < 0.00001). This metanalysis shows a significant OS benefit from concomitant administration of ARPI, docetaxel and ADT in high volume and de novo mHSPC.

## 1. Introduction

Prostate cancer (PC) is the second most frequent cancer diagnosed in men and the fifth leading cause of death worldwide [1]. While, in localized prostate cancer, a personalized treatment approach according to the patient’s class of risk can allow a cure (active surveillance, prostatectomy, radiotherapy +/− ADT), patients with metastatic prostate cancer are often considered incurable [2]. Prostate cancer arises as an androgen-driven disease, and androgen deprivation therapy (ADT) has been the standard of care in metastatic hormone-sensitive PC (mHSPC) since 1940 [3]. ADT induces a response in more than 90% of patients. Despite the high probability of initial response, after a median time of about 20 months, the disease becomes resistant to ADT, with progression to the castration resistant prostate cancer (CRPC) [4]. Recently, the therapeutic strategy of metastatic disease radically changed due to the increased understanding of mechanisms underlying prostate cancer development and progression. It is now well established that most CRPC tumours are not hormone-independent, and that androgen receptor (AR) signalling remains a key driver of resistance and progression in prostate cancer in the phase of castration resistance. Several therapeutic agents have been shown to improve the overall survival (OS) of mCRPC patients. These options include new potent AR pathway inhibitors (ARPI): abiraterone, enzalutamide [5,6,7,8], chemotherapeutic agents: docetaxel, cabazitaxel [9,10,11], radiopharmaceutical therapies: radium 223 and lutetium-177-PSMA-617 [12,13] and poly ADP-ribose polymerase inhibitors (PARPi): olaparib [14]. An essential step in the treatment landscape of metastatic PC occurred in 2015, with the results of the CHARTEED trial [15] showing the efficacy of docetaxel upfront in metastatic hormone-sensitive PC patients. The anticipation in mHSPC of treatments with activity in mCRPC patients has modified the treatment of these patients [16,17,18,19,20,21,22]. These upfront combination therapies aimed to postpone the onset of the castration-resistant phase of the disease and consequently improve OS.

Initial findings showed that in de novo mHSPC patients, the addition of docetaxel to ADT significantly improved OS compared to ADT alone [16,17], especially in high-volume diseases (according to the CHAARTED criteria). More recently, also the addition of ARPI to ADT increased OS. Firstly, two large trials [18,19] demonstrated that adding abiraterone to ADT significantly improved OS. Subsequently, the addition of enzalutamide or apalutamide demonstrated to increase the OS compared to ADT alone [20,21,22].

Recent data provided a further revolution in the treatment landscape of mHSPC [23,24]. In the PEACE-1 study ADT + docetaxel + abiraterone was compared with ADT + docetaxel in 710 de novo mHSPC patients. Triplet therapy increased the OS by about 25% compared to the control arm, especially in high-volume disease with a median OS of 5.1 years vs. 3.5 years [23]. In the second study [24], the phase III ARASENS trial, mHSPC patients were randomized to receive ADT + docetaxel + darolutamide or ADT + docetaxel + placebo. The primary endpoint of OS was met, and the risk of death was significantly lower (about 32%) in the triplet therapy arm than in the placebo arm. Furthermore, the triplet therapy was able to prolong the time to pain progression by about 21% and the time to CRPC by about 61%.

In this rapidly changing treatment landscape, clinicians face crucial challenges; one of the most important is determining the most appropriate therapeutic choice for each patient.

In this context, no head-to-head trials compare ARPI and chemotherapy in mHSPC. An indirect comparison of the efficacy of systemic therapy in combination with ADT was performed by a meta-analysis that did not demonstrate any difference in OS [25]. The only indirect randomized comparative analysis currently available is from the STAMPEDE trial, where the outcome of 566 patients treated with abiraterone or docetaxel was assessed. Although abiraterone was associated with longer failure-free and progression-free survival (PFS), there was no difference in OS and prostate cancer-specific survival [26]. Another crucial challenge will soon be identifying the patients who will benefit most from triplet therapy.

This meta-analysis aims to determine if there is a benefit to combining ARPI, docetaxel and ADT compared with docetaxel and ADT in mHSPC.

## 2. Methods

### 2.1. Study Selection and Data Extraction

MEDLINE/PUBMED and EMBASE searches were performed to identify eligible studies, restricted to phase III, randomised, controlled trials comparing the triplet combination of ARPI, Docetaxel, and ADT to docetaxel and ADT for the first-line treatment of metastatic hormone-sensitive prostate cancer. The proceedings of the European Society of Medical Oncology, American Society of Clinical Oncology, European Society for Radiotherapy and Oncology, and American Society for Radiation Oncology annual meetings were examined for presented abstracts. Based on these criteria, the PEACE-1 and ARASENS studies were selected for our meta-analysis with a percentage of patients in other trials: ARCHES, ENZAMET and TITAN.

### 2.2. Data Extraction

**Figure 1:**
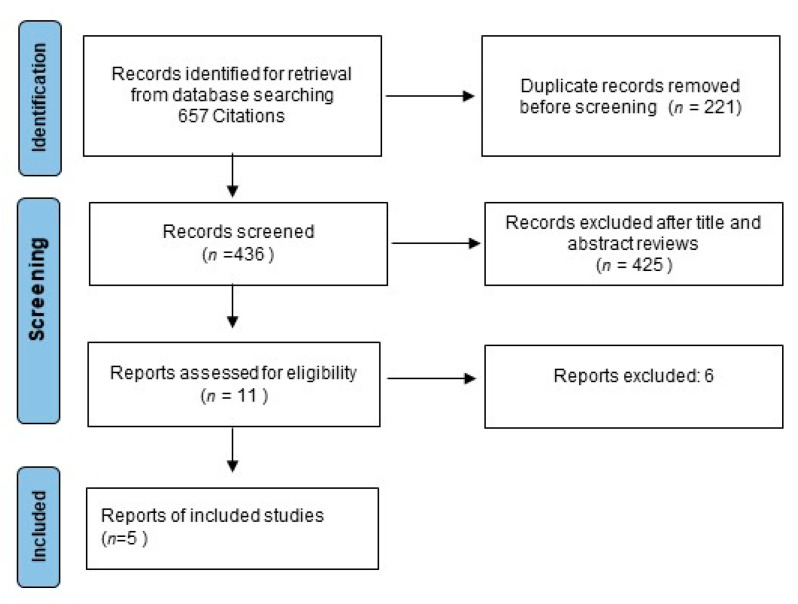
Study flow chart.

Data abstraction was conducted independently by three investigators (MM, DG, FF) following the Preferred Reporting Items for Systematic Reviews and Meta-Analyses (PRISMA) guidance (Figure 1). This review was not recorded on prospective registers; thus, the review protocol was not prospectively available. A list of information about the characteristics of every trial was extrapolated: publication date, first author’s name, sample size, primary endpoints, regimens used, scheduling of triplet therapy, follow-up period, number of outcome events, information about study design, disease-free survival (DFS) definition, OS, subgroup evaluation, quality of life analysis, crossover, if any, and toxicities. Infrequent disagreements among reviewers (overall inter-observer variations <10%) were resolved by discussion.

### 2.3. Statistical Methods

The impact on PFS and OS of treating mHSPC with triplet therapy was measured regarding the hazard ratio (HR). The HR of each study was either extracted directly from the publications or was estimated. Depending on the heterogeneity Χ2-test, the HR estimates were then combined into overall HR using either fixed or random effects models. The fixed-effects model was applied if heterogeneity was not detected (at the 10% significance level). Using the *I*2 coefficient, measuring the percentage of total variation across studies due to heterogeneity rather than chance [27], the heterogeneity of this meta-analysis was quantified. A triplet therapy regimen was deemed beneficial if the HR < 1. Statistical significance was considered if the 95% confidence interval (CI) did not overlap by 1.00 for overall HR.

The association of ARPI, chemotherapy and ADT with toxicities and response rate were calculated in terms of odds ratios, applying the same statistical methods described above.

Begg’s funnel plots were generated to examine potential publication bias related to PFS and OS, and Egger’s regression asymmetry test was performed. A summary survival curve was the most informative finding to resume studies results where survival was used to describe a time-dependent outcome (death or disease recurrence). In this study, we assessed pooled survival probabilities from several single-arm studies using the nonparametric approach reported by Combescure et al. [28].

This approach uses the product-limit estimator of survival to obtain a distribution-free summary survival curve and random effects to model between-study heterogeneity. A matrix of between-study covariances was generated based on the extension of DerSimonian and Laird’s methodology [29,30]. This approach has several advantages over meta-analyses of survival probabilities at a single time point [31]. First, an estimated pooled survival at time t includes all studies, also ended before t, because the conditional survival probabilities before t are estimated with these same studies. Second, this approach makes no assumptions about the shape of survival curves. Finally, there is a guarantee that the combined survival probability will stay the same with time. A *p*-value < 0.05 was regarded as statistically significant for all analyses. The R Statistical Computing Environment (R Foundation for Statistical Computing, Vienna, Austria) was used to complete all analyses and graphics.

## 3. Results

Five RCTs were published between 2019 and 2022, fulfilled the inclusion criteria, and were selected for review. Two randomised phase III studies evaluating the impact of triplet therapy in the treatment of mHSPC have reported DFS and OS. Post hoc analysis of three other trials evaluated the combination of docetaxel to ARPI and ADT; therefore, we used data from these studies.

The key results of the five trials are summarised in Table 1. The PEACE-1 study showed that combining ADT, docetaxel, and abiraterone in de novo metastatic castration-sensitive prostate cancer reduced the risk of death with a modest increase in toxicity. The ARASENS study randomising patients to docetaxel and ADT with or without doralutamide demonstrated a similar improvement in OS. In the other three studies, there was a percentage of patients treated with triplet therapy: 58 pts (11%) in the TITAN trial, 253 pts (44.9%) in the ENZAMET trial, and 103 pts (17.9%) in ARCHES trials. Globally, a total of 2538 patients were included in this meta-analysis: 1270 patients treated with triplet therapy and 1268 with standard treatment as control. The size of the single arm in each study ranged from 58 to 651. The mean patient age was 68.2 years, ranging from 66 to 70. According to the ECOG scale, the performance status defined as “0” went from 64.3 to 77.5%.

The ARASENS (1305 pts) and PEACE-1 (710 pts after the first major protocol amendment) trials were born to evaluate the efficacy and safety of ARPI added to ADT and docetaxel in mHSPC patients. Instead, ENZAMET (205 pts), ARCHES (205 pts), and TITAN (113 pts) trials had some patients treated with triplet therapy, and these data were studied with post hoc analysis.

### 3.1. Progression-Free Survival

**Figure 2:**
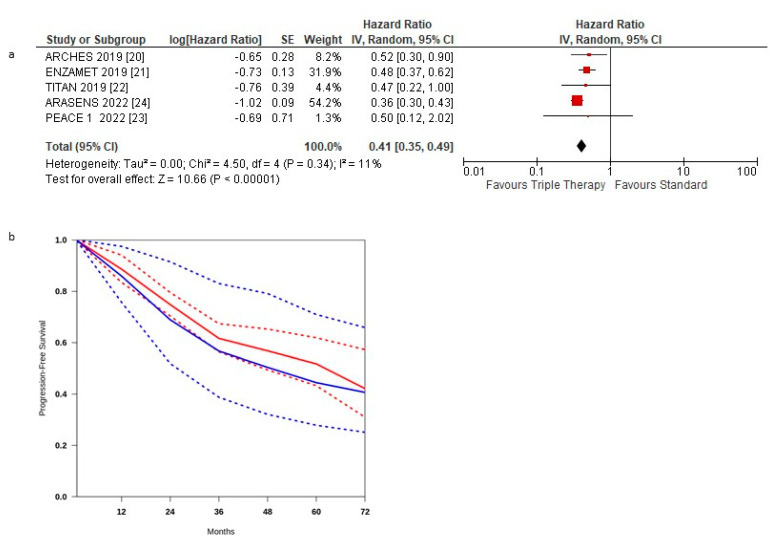
(**a**) Forest plot of progression-free survival hazard ratios obtained using a random model. Studies are arranged by publication year. (**b**) Curves of progression-free survival obtained using the approach of Combescure et al. with random effects. Blue lines represent the summarized disease progression curves and the 95% confidence bands (dashed lines) for control arm. Red lines represent the summarized disease progression curves and the 95% confidence bands (dashed lines) for triplet treatment.

Figure 2a shows the HR for PFS in each trial and the overall analysis. The HRs for PFS of the triplet therapy were compared to the control arms in all trials. The effect of treatment on PFS significantly favoured triplet therapy in all studies, and a statistically significant difference was observed in three studies. Our meta-analysis shows a statistically significant benefit obtained with triplet therapy for mHSPC patients: the pooled estimate of the treatment effect was significant HR 0.41 (95% CI, 0.35 to 0.49) *p* < 0.00001, corresponding to a 59% reduction of the hazard of disease progression for triplet therapy. No significant heterogeneity was observed between the studies (Χ2 = 0.34), *I*2 = 11%. 

The pooled estimate of the treatment effect was significant when using robust analysis. After the omission of the largest trial: ARASENS, the evaluation of the four studies remaining did not lose statistical significance HR 0.75 (95% CI 0.59–0.93) *p* = 0.004.

PFS curves for triplet therapy were extracted from the studies, and the summary survival curves are shown in Figure 2b. The median progression-free survival (95% CI) was 62.4 and 48.4 months, respectively.

### 3.2. Overall Survival

**Figure 3:**
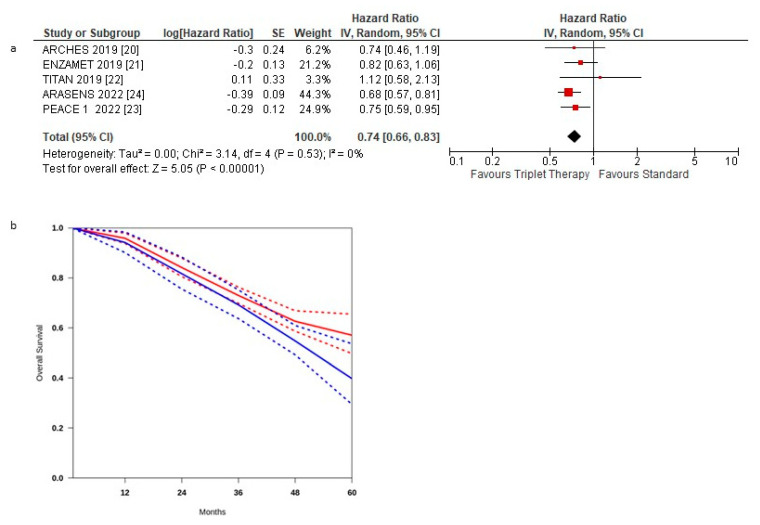
(**a**) Forest plot of overall survival hazard ratios obtained using a random model. Studies are arranged by publication year. (**b**) Curves of overall survival obtained using the approach of Combescure et al. with random effects. Blue lines represent the summarized disease progression curves and the 95% confidence bands (dashed lines) for control arm. Red lines represent the summarized disease progression curves and the 95% confidence bands (dashed lines) for triplet treatment.

Figure 3a shows the HR for OS in each trial and the overall analysis. The treatment effect on OS favoured the triplet therapy in all but one RCTs [23]; a statistically significant difference was observed in only two RCTs [20,21].

The pooled estimate of the treatment effect was significant, HR 0.74 (95% CI, 0.66 to 0.83) *p* < 0.00001, corresponding to a 26% reduction in the hazard of death with triplet therapy. No significant heterogeneity was observed between the studies (Χ2 =0.53), *I*2 = 0%.

The pooled estimate of the treatment effect was significant when using robust analysis. After removing the largest trial: ARASENS, robust analyses showed that all four remaining studies did not lose statistical significance: HR 0.79 (95% CI 0.68–0.93) *p* = 0.004.

Pooling the three post hoc studies, there was no statistically significant benefit, HR 0.83 (95% CI 0.67–1.02) *p* = 0.08. OS curves for triplet therapy were extracted from the studies, and the summary survival curves are shown in Figure 3b. The median overall survival (95% CI) was 52.2 months in the control group and was not reached in the experimental arm.

### 3.3. Overall Survival according to Subgroups

**Figure 4:**
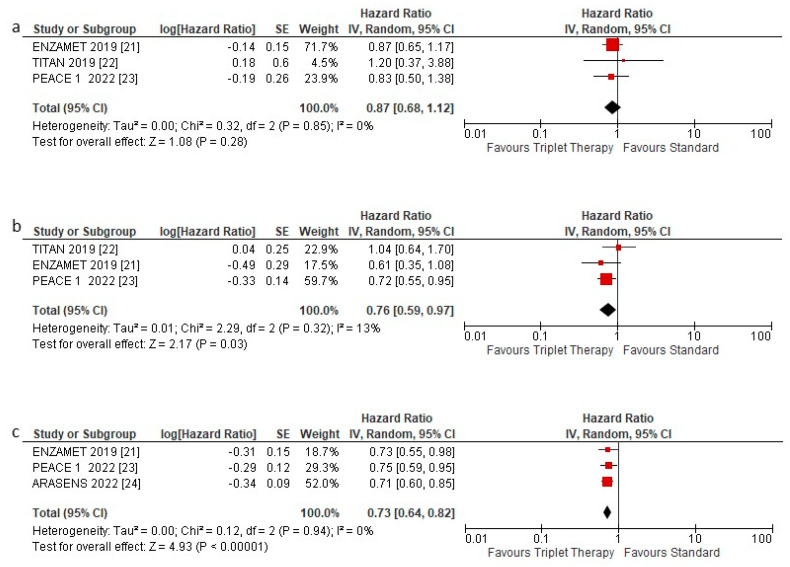
Forest plot of (**a**) overall survival hazard ratios in low volume patients, (**b**) overall survival hazard ratios in high-volume patients, and (**c**) overall survival hazard ratios in de novo patients included in the meta-analysis, obtained using a random model. Studies are arranged by publication year.

OS was assessed according to low- and high-volume disease and de novo metastatic disease. The triplet therapy showed no statistically significant benefit in HSPC patients with low volume metastatic disease HR 0.87 (95% CI 0.68–1.12) *p* = 0.28 (Figure 4a). Instead, a statistically significant benefit was observed in HSPC patients with high volume disease HR 0.76 (95% CI 0.59–0.97) *p* = 0.03 (Figure 4b) and with de novo metastasis, HR 0.73 (95% CI 0.64–0.82) *p* < 0.00001 (Figure 4c).

### 3.4. Safety Profile of Triplet Therapy

**Figure 5:**
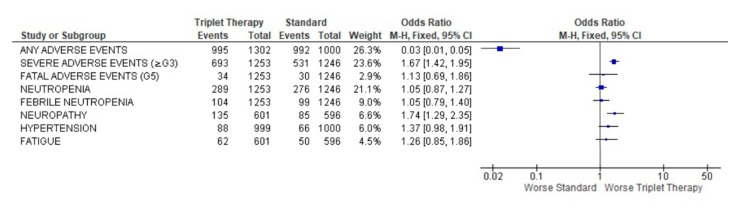
Forest plot of adverse events obtained using fixed effects.

A severe adverse event (≥G3) was described in 1224 patients, 693 in the triplet treatment and 531 in the standard treatment, with OR 1.67 (95% CI 1.42–1.95), *p* < 0.0001. Neuropathy was more frequently recorded in the triplet treatment, 22.5% vs. 14.3%, with an OR of 1.74 (95% CI 1.29–2.35) *p* = 0.0003. No other toxicity rates were statistically significantly different (Figure 5) between the two groups, except, globally, adverse events were more frequent in the control group than in the experimental.

## 4. Publication Bias

**Figure 6:**
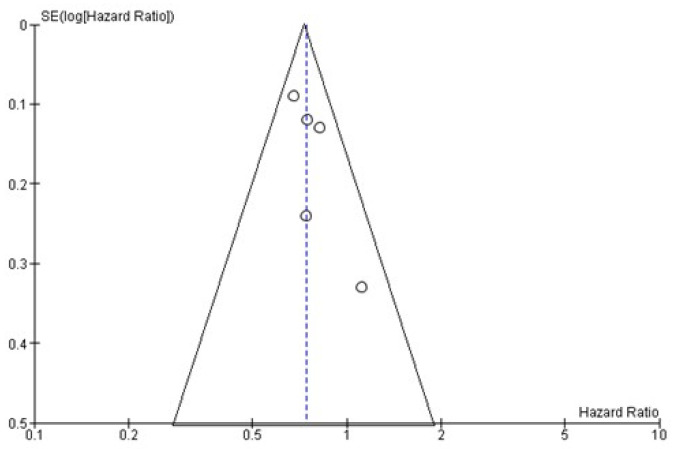
Funnel plot of OS. Symmetry in this graph indicates no publication bias. The circle represents the HR of each single study.

Using the funnel publication bias plot (Figure 6) and Egger’s test for publication bias, the risk of missed or overlooked trials was found to be insignificant (*p* = 0.372).

## 5. Discussion

The results of this meta-analysis demonstrate that adding ARPI to docetaxel and ADT significantly increases OS in high-volume and de novo mHSPC patients. In a rapidly evolving therapeutic landscape, this evidence can help clinicians in the treatment personalization of these patients.

To date, in the absence of any efficacy difference between multiple combination strategies in mHSPC, treatment modality and the toxicity profile, patients’ quality of life (QoL) and patients’ preference have been the determining factors guiding clinicians’ decisions. 

In the STAMPEDE trial, there was a statistically significant difference in the QoL score over two years favouring abiraterone (+3.9 points, 95% CI 0.6–7.1, *p* = 0.021). However, it did not meet the predefined clinically meaningful threshold of ≥4 points [32].

Nowadays, the disease volume represents a crucial clinical factor guiding physicians’ treatment choices. Current guidelines recommend chemotherapy with docetaxel, preferably in high-volume mHSPC, due to the lack of benefit shown by this therapeutic association in low-volume disease (HR = 1.03, 95% CI 0.77–1.38, *p* = 0.8) [33]. Abiraterone, enzalutamide and apalutamide show activity regardless of disease volume and, therefore, can be used in combination with ADT in both low and high-volume patients [34,35].

Beyond the disease volume, the choice of treatment for mHSPC can be influenced by patient comorbidities, toxicity, need for concomitant use of corticosteroids, duration of therapy, patient preferences, costs and reimbursement issues. Recent data from PEACE-1 and ARASENS make the current mHSPC therapeutic work-up more complex. In particular, the ARASENS trial results show that triplet therapy increases OS regardless of disease volume and the presence of synchronous or metachronous metastases. Therefore, triplet therapy could be considered the new standard of care for a relevant portion of mHSPC patients. Consequently, physicians can choose whether to add chemotherapy or ARPI to ADT and which patient can benefit from the triplet therapy. In this context, in the absence of an individual patient data meta-analysis, the results of our study based on abstracted data can help answer this question, allowing a timely synthesis of the available data. The impact of triplet therapy on OS in this disease setting is not unexpected. From a biological point of view, it is widely known that the shift from the hormone-sensitive to a castration-resistant phase of disease follows two progression models: the adaptation and the clonal selection model [36]. In the adaptation model, it is postulated that progression to CRPC is not the result of true androgen independence but is due to the adaptation of cells initially susceptible to ADT to a micro-environment with low levels of androgens. PC cells would acquire drug escape mechanisms allowing them to proliferate even at low androgens levels. Neoplastic cells could induce the production of androgens essential for their survival or change the conformation of AR so that low androgen levels could stimulate it. The upfront use of ARPI enhancing the pressure of ADT on the AR pathway or causing a significant reduction in circulating and tumour levels of testosterone would avoid the onset of these resistance mechanisms, thus delaying the beginning of the castration resistance phase [37]. The clonal selection model postulates that PC is a heterogeneous disease since the hormone-sensitive phase. Before ADT starts, it is characterized by cells with different grades of androgen dependence, so the development of CRPC is due to clonal selection and proliferation of androgen-independent clones. 

In most patients, these two theories coexist. This biological evidence demonstrates that the shift to the castration-resistant phase is due to the coexistence of mechanisms of progression, dependent and independent of AR signalling pathways [36,37].

Docetaxel interferes with both AR-dependent and -independent PC cell progression and survival mechanisms. This drug can induce the inactivation of BCL-2 favouring PC cell apoptosis [38]. Furthermore, docetaxel acts synergically with ADT, interfering with microtubule polymerization and then reducing androgen receptor nuclear translocation and androgen gene expression [37,38].

The biological rationale for the combination of docetaxel and ARPI stems from the potential intra- and inter-tumoral heterogeneity that may be present even at diagnosis and the ability to target subclones with different AR dependence with multimodal treatment. 

The efficacy of triplet therapy, mainly in de novo and high-volume disease, is not surprising. It is recognized that the presence of both a wide metastatic spread and synchronous metastases is related to a more heterogeneous disease and a worse prognosis [19,39,40,41]. The higher aggressiveness of the disease in de novo and the high volume of mHSPC patients would justify the triplet treatment used for its impact in terms of OS and the efficacy in delaying time to CRPC. In the castration-resistant phase, symptoms related to the disease are bound to worsen with harmful interference with patients’ QoL. The administration of triplet therapy in the hormone-sensitive phase of the disease could have another crucial advantage. It could allow therapeutic agents to target both AR-dependent and -independent cells in a more relevant portion of patients. Metastatic HSPC is a less aggressive disease compared to CRPC. CRPC patients are more often symptomatic and characterized by a worse performance than HSPC patients. A relevant percentage of CRPC patients cannot receive chemotherapy [37]. The lack of chemotherapy administration in a highly heterogeneous disease can negatively impact OS and QoL.

However, clinicians must also be careful about the hormone-sensitive disease and side effects related to triplet therapy. Although the side effects associated with triplet therapy are mainly related to chemotherapy, adding an ARPI can increase the risk of toxicity, negatively affecting QoL and, in some cases, OS.

Therefore, clinicians must consider some essential clinical characteristics of patients (performance status, comorbidities, concomitant medications) before considering triplet therapy use. This point is crucial in PC because metastatic PC patients are often over 70 and the side effects of treatments are usually worse in the real-world population compared with those reported in clinical trials. 

Our results can help clinicians identify the patients who most benefit from triplet therapy. However, the results of ARASENS and PEACE-1 studies raised other relevant questions. These two trials showed that triplet therapy increases the OS of mHSPC patients compared to the combination of ADT + docetaxel. However, we have not demonstrated that triplet therapy is more effective than the association of ARPI and ADT. In a recent systematic review and meta-analysis, triplet therapy was not superior over ADT + ARPI doublet therapy (HR 1.22, 95% CI 0.96–1.55 and HR 0.96, 95% CI 0.74–1.23, respectively) [42]. However, there are no trials evaluating triplet therapy versus ADT + ARPI alone. Future studies will have to clarify which patients’ chemotherapy can be avoided because most of the side effects induced by the triplet therapy are docetaxel related. Another relevant question concerns the choice of treatment sequence in patients progressing to triplet therapy. There is no evidence of a possible therapeutic sequence because the results of the studies on the efficacy of triplet therapy are very recent.

In the ARASENS trial, a relevant portion of patients was treated with ARPI as first-line CRPC treatment in both the darolutamide (approximately 50%) and placebo (about 74%) arms [24]. Although it is anticipated that most patients in the placebo arm received ARPI as the first line of therapy in the mCRPC setting, it appears more questionable in patients treated with darolutamide due to the biological and clinical cross-resistance between 2 ARPI used in sequence [43,44]. However, the evidence of cross-resistance between ARPI is limited to the sequence of enzalutamide and abiraterone and vice versa in studies including mCRPC patients. Therefore, the use of a second ARPI, as first-line therapy in the mCRPC setting, could be usable after an ARPI in the mHSPC setting. Data supporting this hypothesis come from a post hoc analysis of the TITAN study. In this analysis, the first life-prolonging therapy was an ARPI (abiraterone or enzalutamide) in 31% of patients. Additionally, in this setting, apalutamide improved PFS [45]. These results, to be interpreted with caution due to their post hoc statistics, hypothesize a possible efficacy for the ARPI-ARPI sequence when the first ARPI is used in the hormone-sensitive setting.

The other treatments available as first-line mCRPC in patients undergoing triplet therapy in mHSPC are radium-223 (only in symptomatic patients with exclusively bone metastases), cabazitaxel (especially in patients with unfavourable clinical prognostic factors), and olaparib (in patients with DDR mutations) and LuPSMA (in patients with positive PSMA-PET disease).

Finally, one of the most intriguing challenges in the treatment choice of mHSPC patients is represented by the identification of biomarkers predicting response to different treatments. In mCRPC, the androgen receptor splice variant 7 (AR-V7) predicts a poor response to ARPI [37,44]. More recently, the introduction of PARP inhibitors and pembrolizumab as therapeutic options in patients with DDR gene mutations or MSI-high have made precision medicine a reality for mCRPC. However, further studies are needed to identify new predictive biomarkers that can help clinicians choose treatment based on the biomolecular characteristics of each patient in mHSPC.

Our study has some limitations. The most important are the differences in trial design and the population of patients enrolled.

The PEACE-1 and ARASENS studies were planned to determine the efficacy of triplet therapy, while the ARCHES, ENZAMET and TITAN studies were not designed with this endpoint. In the ENZAMET, ARCHES, and TITAN studies, the rates of patients treated with docetaxel were 45%, 18%, and 11%. In addition, in the PEACE-1 study, all enrolled patients had synchronous metastases, while in the ARCHES, ENZAMET, TITAN, and ARASENS trials, the percentage of de novo metastatic disease was 66%, 72%, 86%, and 86% respectively. 

Moreover, in the ARASENS study, we lacked data about disease volume, while in the TITAN, ARCHES, PEACE-1, and ENZAMET trials, the rate of patients with high volume disease was 61%, 60%, 57%, and 52%, respectively.

Finally, the timing of docetaxel administration in these studies differed. In the ENZAMET study, patients could be treated with docetaxel concomitantly to ARPI. In contrast, in the ARCHES and TITAN studies, docetaxel chemotherapy had to be concluded before enzalutamide or apalutamide started.

Due to the methodology of this meta-analysis, the results may not be generalizable to new populations and settings. The RCTs were performed by enrolling “healthier” patients. This choice limits the broad application of the results to the “sickest” patients who would potentially be treatable with active treatments. There may be differences in the baseline severity of illness that limit the accuracy of this meta-analysis. Covariates that described patient-level and study-level characteristics were included to control these differences. In our study, patient-level covariates were reported inconsistently across trials, which limited our findings. Hence, the summary results reflect averages rather than individual data because they only reflect variation within studies, not among patients. Furthermore, we missed other potentially significant confounders, which could have impacted our findings.

## 6. Conclusions

The available evidence from this meta-analysis is sufficient to demonstrate a significant impact on OS with the concomitant administration of docetaxel, ARPI and ADT in mHSPC patients with de novo and high-volume metastatic disease. Castration resistance is brought on by ADT’s induction of adaptive alterations in PC cells and the selection of resistant clones. Minimizing this mechanism and delaying the onset of resistance is the clinical key to improving survival in mHSPC. All these novel synergic approaches show significant improvement in outcomes and can be used in clinical practice. Further clinical trials must be developed to increase these results combining local treatment as radiotherapy and therapeutic agents acting on androgen receptor dependent and independent mechanisms of neoplastic progression.

## Figures and Tables

**Table 1:** Study- and patient-level characteristics of the studies in the meta-analysis.

	Type of Study	Experimental Arm	Sample Size	Inclusion Criteria	Median F-Up Months	Mean Age	Gleason Score ≥ 8 (%)	ECOG 0 (%)	High Volume Disease (%)	Bone Metastasis (%)	Visceral Metastasis (%)	De Novo Metastasis (%)
ARCHES [20]	Post hoc analysis RCT	ARPI + Docetaxel + hormosoppression	576	mHSPC	14.4 *	70 *	66 *	77.5 *	63.2 *	84.4 *	4.9 *	72 *
ENZAMET [21]	Post hoc analysis RCT	ARPI + Docetaxel + hormosoppression	503	mHSPC	34 *	69.2 *	58.3 *	72 *	52.3 *	80.7 *	12 *	58 *
TITAN [22]	Post hoc analysis RCT	ARPI + Docetaxel + hormosoppression	113	mHSPC EXCLUDED if visceral met is the only site of metastasis	22.9 *	69 *	67.4 *	64.3 *	62.7 *	100 *	13.6 *	85 *
PEACE 1 [23]	Randomized trial	ARPI + Docetaxel + hormosoppression	710	mHSPC	42	66	76.9	69.8	64.2	79.7	12.4	100
ARASENS [24]	Randomized trial	ARPI + Docetaxel + hormosoppression	1305	mHSPC	nr	67	78.2	71.1	nr	79.5	17.5	86.1

* Data extrapolated from original RCT.
